# Reactive Carbonyl Species: A Missing Link in ROS Signaling

**DOI:** 10.3390/plants8100391

**Published:** 2019-09-30

**Authors:** Jun’ichi Mano, Md. Sanaullah Biswas, Koichi Sugimoto

**Affiliations:** 1 Science Research Center, Organization of Research Initiatives, Yamaguchi University, Yamaguchi 753-8511, Japan; sugimok@yamaguchi-u.ac.jp; 2 Graduate School of Science and Technology for Innovation, Yamaguchi University, Yamaguchi 753-8511, Japan; 3 Department of Horticulture, Bangabandhu Sheikh Mujibur Rahman Agricultural University, Gazipur 1706, Bangladesh; sanaullahbiswas@gmail.com

**Keywords:** abscisic acid, acrolein, auxin, lipid peroxide, oxidative stress, oxylipin, RCS, reactive electrophile species (RES), redox signal, ROS

## Abstract

As reactive oxygen species (ROS) play critical roles in plants to determine cell fate in various physiological situations, there is keen interest in the biochemical processes of ROS signal transmission. Reactive carbonyl species (RCS), the *α*,*β*-unsaturated aldehydes and ketones produced from lipid peroxides, due to their chemical property to covalently modify protein, can mediate ROS signals to proteins. Comprehensive carbonyl analysis in plants has revealed that more than a dozen different RCS, e.g., acrolein, 4-hydroxy-(*E*)-2-nonenal and malondialdehyde, are produced from various membranes, and some of them increase and modify proteins in response to oxidative stimuli. At early stages of response, specific subsets of proteins are selectively modified with RCS. The involvement of RCS in ROS signaling can be judged on three criteria: (1) A stimulus to increase the ROS level in plants leads to the enhancement of RCS levels. (2) Suppression of the increase of RCS by scavenging enzymes or chemicals diminishes the ROS-induced response. (3) Addition of RCS to plants evokes responses similar to those induced by ROS. On these criteria, the RCS action as damaging/signaling agents has been demonstrated for root injury, programmed cell death, senescence of siliques, stomata response to abscisic acid, and root response to auxin. RCS thus act as damage/signal mediators downstream of ROS in a variety of physiological situations. A current picture and perspectives of RCS research are presented in this article.

## 1. Introduction

Plant cells are very rich in reducing agents and redox catalysts. In addition, the above-ground parts contain many pigments. Such conditions are favorable for the production of reactive oxygen species (ROS) via the reduction or excitation of O_2_, and indeed, there are large fluxes of ROS production [1]. If their levels are regulated properly, then ROS are excellent molecules for biological signals; they can turn on biochemical switches temporally in micro-local regions in a cell, and due to their lifetime, the signal is turned off in a short time. 

One of the earliest reports of ROS signaling in plants was on the systemic defense response, demonstrating H_2_O_2_ as a remote signal to bring the oxidative information emitted from the local photooxidized leaves [2]. ROS act as signals, not only in stress responses, but also in hormonal responses and development [3]. It has been shown that the abscisic acid (ABA)-induced activation of respiratory burst oxidase-homolog NADPH oxidases (RBOH) in guard cells contributes to the stomata closure response through the action of H_2_O_2_ [4]. Involvement of ROS has also been suggested in the auxin-induced formation of lateral roots [5] and adventitious roots [6]. ROS are also critical signals for initiating programmed cell death (PCD) in development [7], senescence and pathogen response [8]. Today, there is keen interest in the biochemical processes of ROS signal transduction in plants, but the signaling pathway from ROS to putative sensor/receptor proteins has been poorly elucidated.

**Figure 1:**
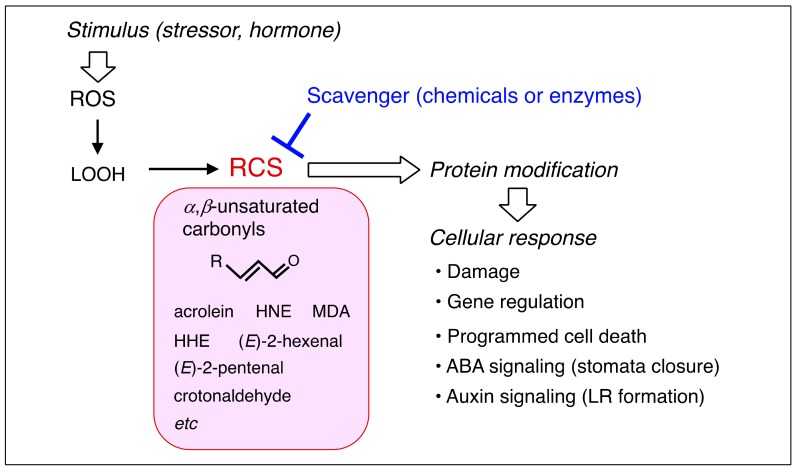
Involvement of reactive carbonyl species (RCS) in reactive oxygen species (ROS) signaling. The involvement is validated by the scavengers’ effects to suppress both RCS levels and a ROS-initiated phenomenon. ABA, abscisic acid; HNE, 4-hydroxy-(*E*)-2-nonenal; HHE, 4-hydroxy-(*E*)-2-hexenal, MDA, malondialdehyde; LR, lateral root.

In this review, we introduce an emerging view that ROS signal is mediated to target proteins by a group of *α*,*β*-unsaturated carbonyl compounds (‘carbonyls’ is a common name of aldehydes and ketones and ‘*α*,*β-* unsaturated’ is for the C–C double bond conjugated with the carbonyl double bond) that are derived from lipid peroxides (LOOH) (Figure 1). We designated this group as ‘reactive carbonyl species (RCS)’ [9]. The established knowledge behind this rather new concept is that ROS are often produced in the close vicinity of membranes in living cells [10] and therefore, membrane lipids are the most immediate and abundant target molecules of ROS. Indeed, analysis of LOOH degradation products in the vitamin E deficient mutant *vte2-1* of *Arabidopsis thaliana* has clearly demonstrated constitutive formation of LOOH and their decomposed carbonyl products in leaves even under relaxed physiological conditions [11]. More than a dozen RCS, including acrolein, 4-hydroxy-(*E*)-2-nonenal (HNE) and malondialdehyde (MDA), have been found in plants [12,13,14,15]. They are alternatively called ‘reactive electrophile species (RES) oxylipins’ [16,17]. We prefer the term RCS, not only because of its brevity, but also because we observe the importance of the carbonyl moiety in the physiological functions of these compounds, while RES comprises a broader range of biological electrophiles, including non-carbonyl ones such as 8-nitro-cGMP and dopamine-3,4-quinone [18]. Methylglyoxal and glyoxal, dicarbonyls derived from sugar metabolism, also have high reactivity and play signaling roles in plants [19], but by our definition, we do not include them in RCS.

RCS can form covalent bonds with proteins to change their functions, and thereby exert various biological effects [20,21,22,23]. This property is suitable for a signal mediator that transmits ROS stimulus to target proteins. It has been known that RCS, when added exogenously to cells, evoke various physiological responses, from gene induction to cell death, both in animals [18] and plants (summarized below). Recent studies combining carbonyl analysis, use of chemical or enzymatic RCS scavengers and observation of plant responses to ROS have revealed that RCS are generated downstream of ROS stimuli and mediate the oxidative signal to the cell (Figure 1). We provide an overview of current research status of RCS in plant physiology. First, we will briefly summarize their production in plants (Section 2). Next, we will summarize the potential actions of RCS from protein modification to toxicity and signaling, by seeing the effects of exogenously added RCS on plants (Section 3) and then examine the physiological roles of the endogenously produced RCS (Section 4). Recent findings of the involvement of RCS in the signaling of plant hormones, i.e., abscisic acid (ABA) and auxin, will be introduced. Last, the regulation of RCS with scavenging enzymes is discussed (Section 5).

Several review articles on the physiological roles of RCS and related compounds in plants are available in what follows, and therefore, the topics discussed therein are treated only briefly in this review. The topics are: the gene regulation by long chain RCS, e.g., phytoprostanes and 12-oxo-phytodienoic acid (OPDA) [16,17]; the stress response of the genes of RCS-scavenging enzymes [24]; and that *β*-carotene oxidation products such as *β*-cyclocitral have the *α*,*β*-unsaturated carbonyl structure and act as a chloroplast stress signal [25].

## 2. Production of RCS in Plants

Critical involvement of ROS in the lipid oxidation and in the degradation of LOOH to various carbonyls was revealed in the 1980s through extensive investigation of the mechanism of cooking oil deterioration at high temperatures [26]. Our current understanding of the mechanism of non-enzymatic formation of carbonyls via lipid peroxidation [27] is based on these in vitro experiments, and it is illustrated in great detail in a recent review [16]. Alternatively, LOOH are produced enzymatically with lipoxygenases (LOXs) and then further processed to various compounds collectively designated as oxylipins, which include jasmonic acid, 12-oxo-phytodienoic acid (OPDA), several RCS and non-RCS carbonyls [28,29,30]. The enzymatic lipid oxidation and oxylipin metabolism in plants have been extensively studied as cellular responses that are relevant to infection and wounding. 

### 2.1. Formation of LOOH

LOOHs are formed from membrane lipids non-enzymatically by ROS in two mechanisms; the radical chain reactions to form hydroperoxyl radical, and the addition of singlet oxygen to form endoperoxide. Both products are converted to hydroperoxides (LOOH) [27]. The hydroperoxyl radical may react with a neighboring lipid molecule to form a peroxide bridge of two lipid molecules [26]. It should be noted that certain species of LOOH are produced only via singlet oxygen (^1^O_2_), but not via the radical mechanism. On the basis of this, the ^1^O_2_-dependent LOOH formation under intense light has been proven with a LC/MS/MS analysis of LOOH species [31].

LOX is a non-heme iron containing enzyme that catalyzes the addition of O_2_ to a polyunsaturated fatty acid. Plant species have multiple LOX isozymes, e.g., *A. thaliana* has six LOXs, which have different specificities of the position of the oxygenated carbon in the substrate [32,33]. The products LOOHs are metabolized rapidly to form various oxylipins, that contribute to plant’s protection against pathogens [34] and herbivores [35,36]. The first step of LOX activation is the association of soluble LOX protein with membrane lipids. When cell structure is disrupted by mechanical wounding, Ca^2+^ ion is released from the sequestered sites and becomes bound to the *N*-terminal domain (PLAT domain) of LOX protein [37]. Binding of Ca^2+^ ion on the PLAT domain causes structural remodeling, so that a hydrophobic area of the LOX can interact with hydrophobic membranes [38]. Along with this, the presence of Ca^2+^ ion chelating agents suppresses the burst of GLV release when *Arabidopsis* leaves are disrupted [39]. LOX activity might be affected by abiotic stressors. Under the condition where 9-LOX and 13-LOX are activated by the elicitor cryptogein, intense illumination on the leaves increased the ROS-mediated LOOH production and eliminated the LOX-dependent LOOH production. This was not due to the competition for substrate lipids because total LOOH levels was higher in darkness [40]. Another work suggested that cadmium activates 13-LOX [41]. Such alteration of LOX activities affects the LOOH composition and may change the RCS composition.

### 2.2. Conversion of LOOH to RCS

Non-enzymatic degradation of LOOH to RCS requires a redox catalyst (e.g., a transition metal ion or a free radical such as lipid peroxyl radical) and a reductant (most kinds of organic molecules such as lipids) [16]. One typical mechanism is the formation of alkoxyl radical (LO^•^) via the reduction of LOOH, followed by the cleavage of the carbon chain of LO^•^ to form organic radicals. The radicals are reduced to form carbonyls [16,24,27]. If a cleaved product aldehyde contains an unsaturated bond, it may be oxidized further by ROS to form a hydroperoxide, which can be converted to hydroxy-aldehyde (e.g., HNE or hydroxy-(*E*)-2-hexenal (HHE)) [42] or oxo-aldehydes (e.g., 4-oxo-(*E*)-2-nonenal and 4-oxo-(*E*)-2-hexenal (OHE)) [43].

**Figure 2:**
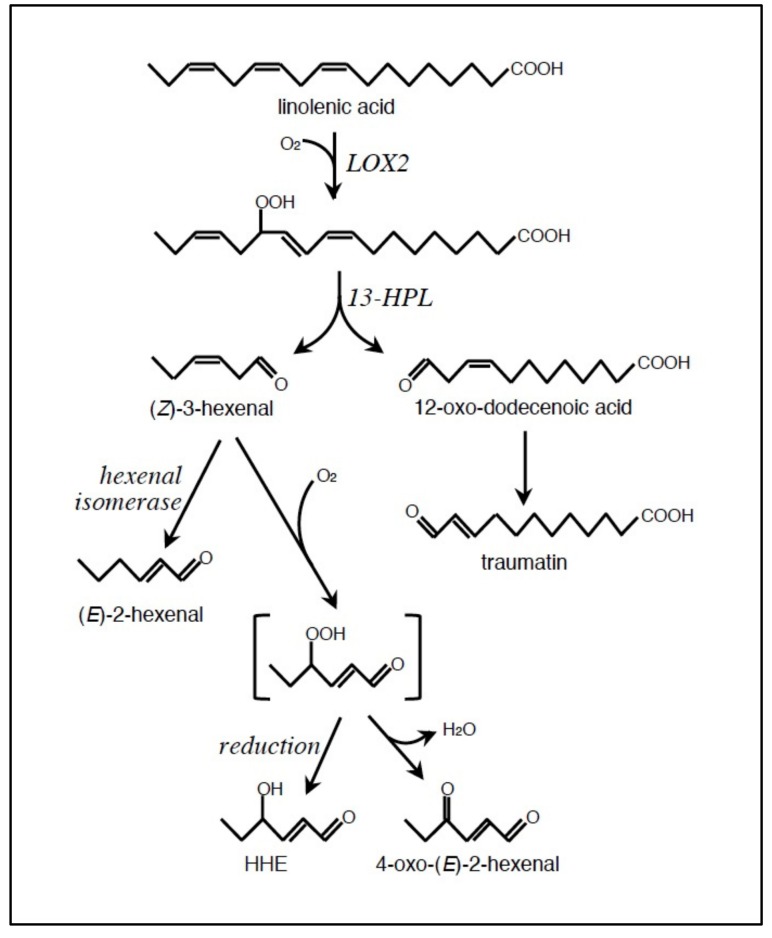
Enzymatic formation of RCS from linolenic acid (18:3) in *A. thaliana*. The oxygenation with LOX2 produces 13-hydroperoxo-octadecatrienoic acid (13-HPOTE) as shown, while with the isozyme 9-LOX, 9-HPOTE is produced. 13-HPOTE is cleaved by the 13-hydroperoxide lyase (13-HPL), while 9-HPOTE by the isozyme 9-HPL, producing C9 aldehydes. When the reaction starts with different unsaturated fatty acids such as linoleic acid (18:2), other sets of aldehydes are formed via a similar series of enzyme reactions.

Alternatively, LOOH are processed with enzymes for various oxylipin carbonyls. In *A. thaliana*, (*Z*)-3-hexenal and 12-oxo-dodecadienoic acid are formed from linolenic acid via the LOX2 and the 13-hydroperoxide lyase (13-HPL) reactions [44] (Figure 2). (*Z*)-3-Hexenal is further isomerized by hexenal isomerase to (*E*)-2-hexenal, an RCS [45,46]. (*Z*)-3-Hexenal potentially produces other RCS, HHE and OHE, by oxygenation [47] but the enzymes involved in this process remain unknown.

LOOH are also metabolized to various oxylipins by several CYP74s, such as CYP74A (allene oxide synthase) for jasmonates, CYP74B (hydroperoxide lyase) for green leaf volatiles, and CYP74D (DES) for divinylethers. It is likely that LOOHs are also processed by other types of enzymes (e.g., reductase, peroxidase, or lipoxygenases) to produce additional oxylipins including short-chain carbonyls [28,47] but the identity of these enzymes remains elusive. Additionally, carbonyls produced by CYP74s will be further processed to produce short-chain carbonyls.

### 2.3. Which Membrane(s) is the Source of RCS?

It is expected that the membranes in the ROS-generating organelles such as chloroplasts and mitochondria are the sources of RCS. The constitutive formation of RCS and non-RCS carbonyls in chloroplasts has been demonstrated in the comprehensive carbonyl analysis of *A. thaliana* leaves, as follows. The *fad7fad8* mutant is deficit of *n*-3 fatty acid biosynthesis in the chloroplast, and has lower linolenic acid (18:3) and higher linoleic acid (18:2) contents in leaves than wild type. In the leaves of the mutant under non-stressed conditions, significantly lower levels of carbonyls than those in the wild type were observed for RCS such as acrolein, crotonaldehyde and HHE and non-RCS carbonyls such as formaldehyde, acetaldehyde and butyraldehyde [48]. Thus, these carbonyls are generated from *n*-3 fatty acids in the chloroplast even in relaxed physiological conditions. Strong illumination on tobacco leaves increased acrolein and (*E*)-2-hexenal levels and caused photoinhibition, suggesting the production of these RCS in the chloroplast [15]. Also, in *Chlamydomonas reinhardtii*, changing light conditions (mixotrophic to photoautotrophic, or strong illumination) caused significant increases in the RCS such as MDA, acrolein, HNE, pentenal, hexenal and non-RCS carbonyls propionaldehyde and hexanal [49,50]. The light-dependent increases suggest that these RCS and non-RCS carbonyls were produced in chloroplasts. In heat stressed *A. thaliana* leaves, the antenna protein LHCII was found to be modified with acrolein, crotonaldehyde and MDA [51], indicating that the thylakoid membrane was the source of these RCS.

When *A. thaliana* cultured cells were exposed to H_2_O_2_, menadione or antimycin A, many proteins in the mitochondrial matrix and membranes were found to be modified with HNE [52], which is clear evidence for the formation of the RCS in mitochondria under oxidative stress. In the leaves of the whole plant under salt stress, HNE modification was increased on the proteins in the cytosol and peroxisome, as well as in the chloroplast and the mitochondrion, suggesting the membranes of each organelle were the sources of RCS [53]. Interestingly, several apoplastic proteins were highly modified (Table 1). Thus, RCS were generated in the plasma membrane as well. 

## 3. Response of Plants to Exogenously Added RCS 

### 3.1. Reaction of RCS with Proteins

**Figure 3:**
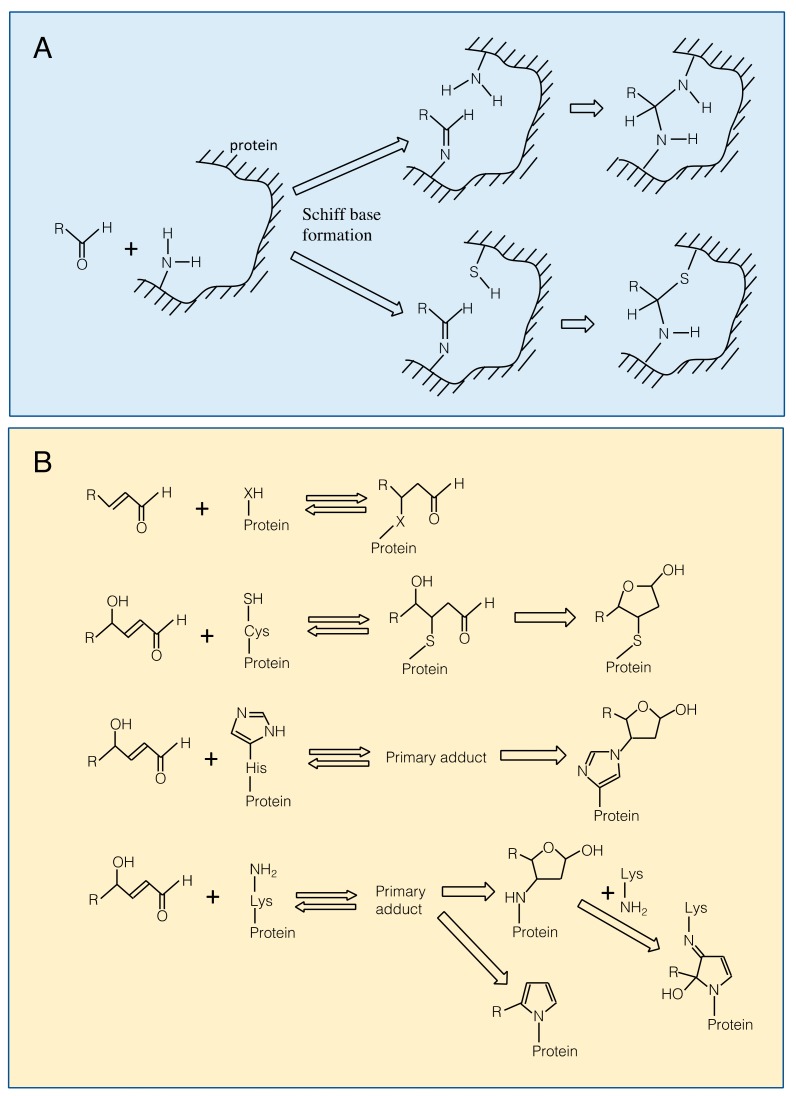
Modification of amino acid residues on a protein with carbonyls. **Panel A**. Schiff base formation on an amino group with an aldehyde and possible secondary reactions. **Panel B. Top**, Michael addition of an RCS to a nucleophilic (-XH) group. X is for sulfur in Cys, ε-amino nitrogen in Lys and imidazole τ-nitrogen in His residues. **From 2nd to bottom**, Michael addition of a 4-hydroxy-(*E*)-2-alkenal to Cys, His and Lys residues. Primary adducts may undergo cyclization.

Carbonyl compounds are electrophiles and can react with nucleophilic groups on biomolecules to make a covalent bond [12]. Typical reactions are Schiff base formation and Michael addition. Schiff base is formed between a carbonyl group and an amino group through dehydration (Figure 3A). This reaction proceeds fast at pH 5, but slower at lower or higher pH conditions. The formed Schiff base can be hydrolyzed under a highly acidic condition. When a thiol group or an amino group reside nearby the Schiff base, the secondary reactions may proceed to form a complex structure [12], which may contribute to the formation of cross-link between proteins. Michael addition is the reaction of the electrophilic β carbon of an RCS molecule with the thiol sulfur, amino nitrogen, or the imidazole τ-nitrogen atom to form a covalent bond (Figure 3B). They also react with the guanine base of nucleic acids, leading to a mutation [20]. The Michael reaction with a thiol is reversible. The equilibrium constant of the reaction between GSH and an RCS differs greatly, depending on the type of RCS molecules [54].

Michael addition of an RCS to a protein adds a carbonyl moiety on the protein (protein carbonylation), while Schiff base formation of RCS with a protein does not. Carbonyl moieties on a protein can also be formed by ROS directly at Trp, His, Tyr, Met and Cys residues [55,56]. Protein carbonylation has been conventionally regarded as an indicator of protein oxidation in plants, but it should be noted that a considerable portion of the detected proteins as ‘oxidized proteins’ are modified with RCS [53].

The primary Michael adducts, or protein carbonyls, can proceed to secondary reactions with another nucleophilic group, to produce irreversible structural changes on the protein (Figure 3B). The primary Michael adduct between a nucleophile and 4-hydroxyl-2-alkenals, e.g., HNE and HHE, can undergo cyclization. The cyclic secondary products may further react with another nucleophilic group [20]. These reactions can lead to the formation of cross-links between proteins.

Effects of RCS modification on the protein function differs by the kinds of RCS and proteins. The RCS includes carbonyls with various structure; different carbon chain lengths, number of unsaturated bonds, and the extent of oxygenation [9,24]. Hence, every RCS has different properties such as polarity, hydrophobicity/hydrophilicity, solubility, and volatility [57]. This variety gives distinct RCS molecules different reactivity with proteins, implying that each species can bring distinct information via specific recognition by the targets and scavenging enzymes. For example, HNE and 4-oxo-(*E*)-2-nonenal (ONE), two C_9_-RCS differing only by the extent of oxidation at the 4th carbon, show a great difference in the reactivity with GSH; the second-order reaction constant of the former is 100-fold smaller than that of the latter [58]. Interestingly, these two RCS react with the redox sensor protein mitoNEET differently, but in a manner not simply deduced from the reactivity discussed above; ONE is bound to Lys55 specifically, while HNE adds to Lys and His residues broadly on the protein [59]. Several isozymes of glutathione transferase (GST) Tau class recognize acrolein and HNE as substrates, but there are ones that accept acrolein only [60,61]. These examples suggest the difficulty in generalizing the mode of interaction between RCS and their target proteins.

Lists of proteins that are affected by RCS are available for animal proteins [22,23,62], but only a few plant proteins have been investigated, as described below. In general, when an RCS acts as a signal, the (putative) target protein probably will gain the function, so that modification of a small portion of the total population of the target might trigger the next signal. In case of damage, inactivation of an RCS target protein will affect a cellular process immediately if the activity of that protein limits the metabolic or signaling pathway involving it. Extensive modification of a protein will also facilitate cross-linking with other proteins, leading to aggregation unless the modified proteins are proteolytically removed (described below). Under severe and prolonged oxidative stress, larger populations of broader range of proteins will be inactivated, leading to extensive deterioration of cell functions.

In Table 1, plant proteins that are sensitive to or modified by RCS are listed. Addition of HNE to isolated mitochondria caused sensitive inactivation of lipoate enzymes such as H-subunit (SU) of glycine decarboxylase complex (GDC) [63] and alternative oxidase [64]. Addition of RCS, especially acrolein, to chloroplasts caused a rapid consumption of GSH, followed by the inactivation of phosphoribulokinase (PRK) preferentially, then fructose-1,6-bisphosphatase, glyceraldehyde-3-phosphate dehydrogenase, leading to the loss of the CO_2_ fixation ability [13]. By a proteomic analysis of *A. thaliana* cultured cells treated with oxidative agents such as H_2_O_2_, menadione and antimycin A, subsets of inner membrane proteins and matrix proteins (totally 31 different proteins) were identified as HNE targets because they were modified by this RCS to greater extents under the stress [52]. In heat-stressed spinach leaves, OEC33 protein in photosystem II (PSII) was modified with MDA and acrolein, while in *A. thaliana* leaves the antenna LHCII protein was sensitively modified with MDA in heat stress [51]. Mano et al. [53] analyzed the RCS-modification of soluble proteins in leaves from salt stressed *A. thaliana* plants. As detected with specific antibodies, protein modification with HNE, 4-hydroxy-(*E*)-2-hexenal, acrolein, crotonaldehyde and malondialdehyde increased in leaves with the progress of the salt-stress treatment. In addition, the acrolein- and crotonaldehyde-modifications were increased significantly even under less severe stress conditions, in which there was no apparent tissue injury or the photoinhibition of PSII. The band pattern of Western blotting suggested these different RCS targeted a common set of proteins. With a quantitative proteomic analysis after immuno-affinity trapping of the HNE-modified proteins, 17 distinct proteins were identified as sensitive targets. Interestingly, these target proteins were distributed to various cellular compartments, i.e., cytoplasm, peroxisome, chloroplast, nucleus and even apoplast. Addition of acrolein to tobacco bright yellow-2 (BY-2) cultured cells or the cell extract activated caspase-1-like protease (C1LP) and caspase-3-like protease (C3LP) activities [65]. Results of these experiments with different samples and treatments commonly suggest that certain subsets of proteins are sensitively modified with RCS under stress conditions. In other words, intracellular RCS do not necessarily react with broad range of proteins.

The fate of RCS-modified proteins has not been significantly investigated, both in animals and plants. In general, ‘damaged’ proteins should be immediately degraded before extensive modification leads to the formation of aggregated proteins, which may escape degradation and deposit in cells to cause detrimental effects. Mildly modified proteins are degraded by proteasomes [67]. In mice, the 20S proteasome has been shown to be responsible for the elimination of most oxidized proteins [68]. In *A. thaliana* also, the 20S proteasome appears to be responsible for the degradation of oxidized proteins, while the 26S proteasome takes care of misfolded proteins [69]. In mitochondria, ATP-dependent proteases such as Lon protease has been recognized as key proteases for the removal of oxidized proteins in mammals and yeast, but not in plants [70]. These studies have investigated “oxidized” or “carbonylated” proteins collectively and have not distinguished ROS-mediated and RCS-mediated modification on proteins. There is a report that the degradation HNE-modified protein is catalyzed by cathepsin G in rat [71], but corresponding facts for plants are not available. It should be noted that the modification of proteins with certain kinds of RCS such as MDA are reversible [72]. Thus, it is possible that a transient increase in the RCS concentration modifies the target protein(s) and afterwards the RCS is released from the protein, and then scavenged by certain enzymes (described below). In this case the target protein can be recruited to its physiological role although there has been no experimental data available for demonstrating such dynamics. 

### 3.2. Cytotoxicity

RCS added from outside of the cells are toxic to plants when their concentrations are high. Reynolds [73] evaluated the toxicity of various carbonyls for their ability to inhibit germination of lettuce seeds and demonstrated that RCS have relatively higher toxicity than non-RCS carbonyls. Exposure of *A. thaliana* plants to acrolein or methylvinylketone (MVK) as volatiles (10 *μ*mol/L air) caused a decrease in PSII activity in 15 h [74]. Similarly, (*E*)-2-hexenal (100 *μ*mol/L air) significantly decreased PSII activity in 5 h [75]. When a 20 *μ*M aqueous solution of HNE was infiltrated into *A. thaliana* leaves, PSII was inactivated in 15 h [74] and higher concentrations at mM levels caused necrosis [76]. Aqueous 10 *μ*M HNE inhibited root elongation in tobacco plants [14].

At subcellular levels, the addition of HNE to isolated mitochondria inactivated respiratory metabolism in the matrix [77]. In this case, the most sensitive targets were lipoate enzymes as described above [63]. When isolated chloroplasts were treated with acrolein in darkness, their photosynthetic activity was lost, but photosynthetic electron transport chain was insensitive [13]. On the other hand, the addition of acrolein to the *Synechocystis* cells under light inactivated PSII and caused growth inhibition. The inactivation mechanism is accounted for by the combined effect of acrolein and hydroxyl radical, which was generated under light [78]. 

### 3.3. Signaling Effects of Exogenously Added RCS

Exogenous application of RCS at low levels to whole plants can induce arrays of defense genes. In *A. thaliana* seedlings exposed to (*E*)-2-hexenal, a C6 volatile RCS that can be produced enzymatically in response to pathogen attack, defense response genes including phenylpropanoid synthesis enzymes were induced [34,79]. Low levels of MDA strongly upregulated many abiotic/environmental stress-related genes such as *ROF1* and *XERO2* in *A. thaliana* [72]. When acrolein and MVK were infiltrated to *A. thaliana* leaves, pathogenesis-related genes such as *HEL*/*PR4* were activated [74]. Fumigation of *A. thaliana* plants with RCS induced a group of heat shock response genes [80]. The putative RCS receptor involved in this response should have a specificity to certain types of RCS because this signaling effect was observed for RCS of carbon chain length 4–8, but not for the C3 RCS acrolein. Addition of acrolein, HHE and HNE to *A. thaliana* caused increases in the activity of various ROS scavenging enzymes such as catalase and ascorbate peroxidase [81]. Interestingly, the extreme halophyte *Eutrema parvulum*, a close relative to *A. thaliana*, did not respond similarly to these RCS; the enzyme activities were not increased. This suggests that the RCS perception systems or RCS scavenging selectivity and capacity in halophytes are different from those in glycophytes.

Addition of acrolein to *C. reinhardtii* cells up to 600 ppm increased the GSTS1 content and improved cells’ tolerance to ^1^O_2_. On the basis of transcriptomic analysis, it is suggested that acrolein can mediate the gene expression signal triggered by ^1^O_2_ [50].

OPDA, a phytohormone and a precursor to jasmonic acid (JA), has a cyclopentenone structure and is an RCS. When applied to *A. thaliana* plants exogenously, OPDA induces a set of defense genes that is distinct from those induced by JA [82], and represses the expression of those involved in cell cycle regulation and cell growth [83]. This OPDA signal is recognized by the TGA transcription factors [84]. OPDA can be bound also to cyclophilin 20-3 and alter the protein’s ability to trigger the formation of cysteine synthase complex, and thereby affect the redox homeostasis [66].

## 4. Evidence for Physiological Functions of Endogenously Produced RCS 

The experiments above have demonstrated the capability of RCS to affect cellular metabolism, gene expression and fate of the cell, but physiological relevance of these results needs to be considered carefully, because the increase of intracellular RCS levels made by exogenous addition might be too high or too abrupt. The physiological functions of endogenously generated RCS, therefore, should be validated with the following observations. (1) After ROS stimulus to cells, the RCS level should be increased, and (2) scavenging of intracellular RCS should suppress the ROS-induced response (Figure 1).

### 4.1. Cytotoxicity of Endogenously Generated RCS

If RCS are responsible for the damage by these stressors, their intracellular levels should be increased before detectable damage develops, and in the tolerant plants that overproduce RCS-scavenging enzymes, such increases should be smaller. To analyze various RCS potentially generated in cells, we have employed the method of dinitrophenyl hydrazine (DNPH)-derivatization followed by a reverse-phase HPLC analysis, in which both RCS and non-RCS carbonyls are determined [85]. A detailed protocol of this comprehensive analysis method, including the preparation of DNPH-derivatized carbonyl standards is available [86]. Comprehensive carbonyl analysis has revealed that plant tissues contain dozens of different carbonyls constitutively under relaxed physiological conditions [14,15,48]. Major species include non-RCS carbonyls formaldehyde, acetaldehyde and acetone, and their levels were in a range of sub-*μ*mol per g fresh weight, corresponding to sub-mM level on a simple assumption that they are distributed homogenously in cells. RCS such as (*E*)-2-hexenal, (*E*)-2-pentenal, crotonaldehyde, HNE, HHE and acrolein were also found as constitutive species at *μ*M levels [14,15].

Changes of carbonyl content was first analyzed in transgenic tobacco that overproduces 2-alkenal reductase (AER) [14,15]. AER catalyzes the reduction of *α*,*β*-unsaturated bond of RCS with NADPH [87], and the AER-overexpressing (AER-OE) tobacco plants showed tolerance to photooxidative stress [76]. In leaves of both wild type and AER-OE tobaccos, 16 distinct carbonyl compounds were detected, with a dozen unidentified species. At 30 min illumination of photoinhibitory light, when there was no apparent difference in the photoinhibition between wild type and AER-OE lines, there was already significant differences detected in the increase of acrolein, (*E*)-2-pentenal and (*E*)-2-hexenal [15]. These RCS were increased 2–3 fold in wild type, and in AER-OE plants, their increases were significantly lower. The tolerance of AER-OE plants thus was ascribable to the suppression of these RCS. The involvement of RCS in root injury due to aluminum (Al) stress has been also demonstrated as follows [14]. Al ion is preferentially accumulated in the elongation zone of a root, and induces the production of ROS in mitochondria. Accumulation of carbonyls and cell death were observed in the zone, leading to the inhibition of root elongation. AER-OE tobaccos suffered less Al stress, and the increases of RCS (e.g., acrolein, HNE, HHE, (*E*)-2-heptenal) were significantly smaller than wild type. Because the Al treatment caused the increase in ROS to the same level both in AER-OE plants and in the wild type, the Al-tolerance of AER-OE line was unequivocally attributable to the suppression of carbonyl levels.

It should be noted that certain non-RCS carbonyls, e.g., formaldehyde and acetaldehyde, that were contained at high levels under relaxed physiological conditions were also increased, and reached sub-mM to mM levels, one order of magnitude higher than those of highly toxic RCS such as acrolein and HNE. The Al treatment increased the level of HNE by 1.2 nmol g FW^−1^, whereas that of MDA by 7.3 nmol g FW^−1^ [14]. Considering that the reactivity of MDA is one-tenth that of HNE [88], we can evaluate the damaging effect of MDA and HNE in vivo were comparable. Similarly, the contribution of formaldehyde and acrolein to the damage would be 1:4, on an assumption that the former is 400 times weaker than latter [73]. Thus, RCS and non-RCS carbonyls may collectively act as injuring agents.

In the leaves of *A. thaliana* plants under salt stress, there were significant increases in the protein modification, prior to visible lesion on the leaves, with various RCS, e.g., acrolein, crotonaldehyde, HNE and HHE [61]. It should be noted that the modification with acrolein and crotonaldehyde preceded, but with MDA appeared at significantly later stage. Thus, early rising RCS are likely to play more critical roles as a cause of tissue damage. MDA may represent the developed stage of oxidative stress.

RCS production can be altered also by genetic modification of fatty acid composition. Cyclamen leaves are sensitive to heat, but the transgenic plants with high saturated fatty acid contents show heat tolerance. When plants were heat-treated, there was a large transient increase in (*E*)-2-hexenal in wild type, but none in the tolerant transgenic lines. Subsequently, as the visible lesions in the leaves develop, acrolein and MVK were increased only in the wild type [89]. These RCS might directly damage the leaf tissue or trigger PCD, as described below.

These reports, showing close correlation between the RCS levels and extent of tissue damage, have successfully demonstrated that endogenously generated RCS are critical causes of cell injury under oxidative stress. 

### 4.2. Signals Transmitted by Endogenously Generated RCS

#### 4.2.1. Programmed Cell Death (PCD)

The ROS-induced initiation of PCD in tobacco BY-2 cells [88] satisfies the following three criteria for the involvement of RCS as ROS signal mediators. (1) *ROS stimulus should increase RCS levels in the cell*: Treatment of the tobacco BY-2 cells with 1 mM H_2_O_2_ went to PCD in 5 h. In 2 h, prior to the appearance of PCD symptoms, the levels of several RCS, e.g., acrolein and HNE were increased twofold. (2) *Addition of RCS should result in ROS-stimulated responses*: These RCS, when added to BY-2 cells, induced PCD symptoms. (3) *Scavenging of RCS should suppress the response of cells to ROS*: Chemical scavengers for carbonyls eliminated both the RCS increase and PCD, while they did not affect the enhancement of intracellular levels ROS and LOOH. 

Among the RCS increased by the H_2_O_2_ treatment, MDA and acrolein increased the most (2.0–2.5 nmol g FW^−1^). Exogenous addition of acrolein and HNE as low as 0.2 mM caused PCD [88]. Considering that MDA is tenfold weaker than HNE [54], the primary RCS responsible for the H_2_O_2_-induced PCD would be acrolein. Indeed, the addition of acrolein to the cells cause the activation of C3LP and C1LP activities in 10 min, as was observed for the addition of H_2_O_2_. The mechanism of PCD initiation by RCS can be accounted for the direct activation of C3LP by RCS [65]. The signaling role of RCS to initiate PCD was suggested also for a whole plant. AER-OE tobaccos suffered significantly lower PCD than wild type in root epidermis after stress treatment with H_2_O_2_ or NaCl [88]. 

#### 4.2.2. Senescence of Siliques

Similar to PCD, the senescence of silique in *A. thaliana* involves RCS as follows [90]. Arabidopsis aldehyde oxidase 4 (AAO4), catalyzing the oxidation of acrolein and various non-RCS carbonyls, is specifically expressed in siliques. The *ALDEHYDE OXIDASE 4* knocked-out (AAO4 KO) *A. thaliana* mutant showed facilitated senescence of siliques in darkness and concomitant accumulation of acrolein and MDA to levels higher than wild type. Exogenous application of RCS such as acrolein and HNE to siliques caused senescence of the organ in the mutant, but not in wild-type.

#### 4.2.3. ABA Signaling for Stomata Closure

Recently, the function of RCS to mediate ROS signal in hormone responses has been demonstrated. When ABA act on the guard cells to cause stomata closure, the NADPH oxidases on the plasma membrane are activated and the resulting H_2_O_2_ mediate the ABA signal [4]. It was found that several types of RCS such as acrolein and HHE were increased in epidermis of tobacco leaves on ABA addition [91]. Transgenic plants overexpressing AER did not respond to ABA, with respect to both the RCS increases and stomata closure in response to ABA, drought and H_2_O_2_. Furthermore, RCS addition to wild-type epidermis induced stomata closure response, and stomata reopened when RCS was washed out. These results altogether indicate that ABA stimulates the formation of H_2_O_2_ in the guard cells, and thereby increased RCS mediate the signal for closing stomata. The signaling action of RCS was dependent on the activation of the plasma membrane Ca^2+^-permeable cation channels, indicating the RCS react with a component(s) upstream of the channel activation [92]. 

#### 4.2.4. Auxin Signaling for Lateral Root Formation

We recently published a report for the involvement of RCS in the auxin signaling for lateral root (LR) formation [93]. It has been shown that ROS can enhance the auxin-dependent formation of LR [5]. Our carbonyl analysis showed that several RCS, e.g., HNE and crotonaldehyde and non-RCS, e.g., formaldehyde and butyraldehyde were increased in roots in 5 h after auxin treatment, prior to apparent LR formation. Addition of these RCS promoted the degradation of an Aux/IAA repressor, drove several auxin-responsive genes, and increased the LR formation. These results clearly indicate that RCS, produced downstream of ROS, reinforced the auxin signaling for LR formation. The action mechanism of RCS is presumed as facilitation of the formation of TIR1-Aux/IAA-auxin complex via the modification of the involved protein(s) [93].

#### 4.2.5. Retrograde Signaling of *β*-Carotene Oxidation Products

Oxidative degradation of *β*-carotene results in the formation of *β*-cyclocitral, an *α*,*β*-unsaturated carbonyl. It acts as a retrograde signal agent generated in chloroplasts to induce a set of genes for defense against stress [94]. Although this carbonyl is not from membrane lipids, it shares common chemical properties with lipid-derived RCS, and may have common target/receptor proteins. Because lipid-derived RCS production in chloroplasts is also increased by environmental stressors, they may also work as retrograde signals, if they diffuse out of the plastid. 

These experimental facts collected, as described above (Section 4.1. and Section 4.2) indicate the critical importance of RCS as the mediators of oxidative signal and oxidative injury in plants under various physiological situations. Thus, RCS provide a missing link between ROS and target proteins in the oxidative signaling.

## 5. RCS-Scavenging System

RCS have a ‘double-edged sword’ character, as do ROS, i.e., they act as essential signals when their levels and localizations are limited, but can exert deleterious effects on a cell when their levels rise beyond the cell’s control. Plant cells therefore have to harness RCS with multiple mechanisms, as they do for ROS. There are non-enzymatic scavengers and scavenging enzymes for RCS.

### 5.1. Small Molecule Scavengers

A thiol group forms a Michael adduct with RCS. Among common small biomolecules, cysteine is the fastest to react with RCS, and then is the reduced form of glutathione (GSH) to produce an adduct (‘GSH conjugate’) [54]. The adduct formation between GSH and RCS is reversible, and the equilibration constant is different by the kind of RCS; for example, at pH 7.4, at the equilibrium between GSH and acrolein, the free GSH fraction is 1.2% and for HNE it is 8.5%. For crotonaldehyde, in contrast, it is 46.8% [54]. GSH is the first line of defense against acrolein. The addition of acrolein consumes GSH in tobacco BY-2 cells or isolated chloroplasts very rapidly, while ascorbate consumption is much slower [13,65]. Higher GSH level in plant cells thus contribute to stress tolerance by suppressing both ROS and RCS levels [95]. This GSH conjugation with acrolein is most likely mediated by glutathione transferase (GST) isozymes that have specificity for acrolein (described below). When GSH-RCS conjugates are formed in cells, they will be metabolized in a pathway to degrade glutathione-conjugates [96,97] although the exact degradation route of GSH-RCS conjugates has not been elucidated in plants.

Any small nucleophilic compounds comprising thiol-, amino- and imidazole groups are capable of scavenging RCS when their concentrations are high enough (for chemistry, see Figure 3). Carnosine, a dipeptide contained in muscles and brains, reacts with an aldehyde via the Schiff’s base formation between the amino end of *β*-Ala and the aldehyde moiety [98,99]. There have been no reports for plants to have carnosine and related peptides. RCS can be reduced by NADH and NADPH non-enzymatically as well as enzymatically (described below). Polyphenols such as phloretin (found in tea and apple) [100] and pelargonidin (brown rice) [101] also can act as RCS scavengers; they can bind RCS. The contribution of these small molecules to the scavenging is determined by their concentrations in the cell.

### 5.2. Enzymes

**Figure 4:**
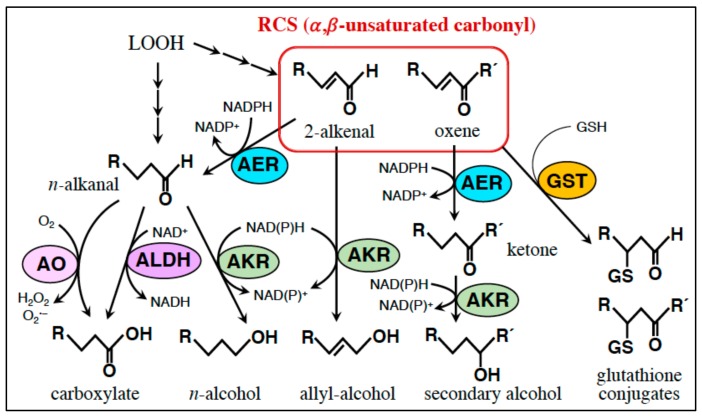
Reactions of RCS scavenging enzymes. See text for abbreviations.

Five types of enzyme reaction have been known for scavenging/detoxifying RCS and carbonyls in plants (Figure 4): (1) The reduction of the *α*,*β*-unsaturated bond in an RCS molecule using NAD(P)H as the electron donor. This is catalyzed with AER and alkenal/one oxidoreductase (AOR). (2) The formation of a glutathione adduct of RCS, catalyzed with GST. (3) The reduction of a carbonyl moiety to an alcohol using NAD(P)H, catalyzed with aldo-keto reductase (AKR). (4) The oxidation of an aldehyde to a carboxylic acid using NAD^+^ as the electron acceptor. This is catalyzed with aldehyde dehydrogenase (ALDH). (5) The oxidation of an aldehyde using O_2_ as the electron acceptor is catalyzed with aldehyde oxidase (AO). Each enzyme group comprises multiple isozymes in one plant species and each isozyme has a different substrate specificity from another isozyme [24]. 

To understand the whole picture of RCS regulation mechanisms in plants, we need to collect the biochemical and physiological knowledges about each isozyme, as follows. (1) The specificity of an isozyme for RCS and non-RCS carbonyls. Although accumulated data are not comprehensive, rather just fragmental [24], recent results have given us some insights. (2) The carbonyls scavenged by each isozyme in vivo. An isozyme in cells can scavenge a carbonyl species only when they meet in a same compartment. The tissue distribution, expression pattern and intracellular localization of various enzymes can be deduced from genomic and transcriptomic analysis, but it is very difficult to determine the localization of distinct carbonyls. Carbonyl analysis of transgenic plants gives indirect information to help our understanding of the carbonyl scavenging system in the cell. There are recent review articles about AKR [24,102] and ALDH [103,104,105] in plants, and in this review, we will focus on AER, GST and AO.

#### 5.2.1. AER and AOR: Reduction of the *α*,*β*-Unsaturated Bond.

AER from *A. thaliana* (AtAER, encoded by the gene *At16950*) [76,87] and AOR [106] catalyze the NAD(P)H-dependent reduction of the carbonyl-conjugated C–C double bond specifically and produce the corresponding saturated carbonyl (Figure 4). Both enzymes prefer NADPH to NADH [87,106]. Neither of these react with saturated carbonyls. AtAER, belonging to the leukotriene dehydrogenase (LDH) branch in the middle chain dehydrogenase/reductase superfamily [107], is a cytosolic protein [76], while AOR, belonging to another branch of the same superfamily, is chloroplastic [106].

AtAER recognizes aliphatic RCS of carbon chain length 3–12 as an electron acceptor, preferring longer chain RCS with higher catalytic efficiency [76,87]. It catalyzes the reduction of 7–8-double bond of phenylpropanals such as *p*-coumarylaldehyde and coniferylaldehyde, as does phenylpropanal reductase from loblolly pine [108], an ortholog of AtAER, but incompatible with the cyclic enones such as OPDA and cyclohex-2-en-1-one [87]. An ortholog in raspberry is involved in biosynthesis of raspberry ketone, the main flavor in the berry [109]. *A. thaliana* genome encodes 11 paralogs of AER [87], which appear to have various substrate specificities. For example, AtAER and its isozyme encoded by *At16950* have 92% identity of the amino acid sequences and show similar levels of the specific activity as a quinone reductase, but their substrate specificity for RCS are contrasting; the former recognizes a broad range (C_3_-C_12_) of RCS (both 2-alkenals and oxenes), while the latter recognize HNE only with a very low activity (3% of the former) (Satoshi Sano, personal communication). 

Overexpression of AtAER in tobacco suppresses the stress-induced increase in a broad range of oxylipin carbonyls and brings various outcomes. Transgenic AER-OE tobacco, as compared with wild type, had alleviated damage due to intense light, methylviologen [76] and aluminium [14]. PCD in root epidermis under salt stress was suppressed in AER-OE plants [88]. In *A. thaliana*, when driven by the controlled overexpression system, AER brought salt tolerance in seeds and seedlings [110]. In the AER-OE tobaccos, two interesting facts were observed as follows. First, overexpression of AER caused the suppression of the ROS-induced increases in the levels both RCS and non-RCS carbonyls [14,88,91]. The suppressed non-RCS carbonyls included LOOH-derived species such as *n*-hexanal and (*Z*)-3-hexenal, as well as very short carbonyls such as formaldehyde, acetaldehyde and acetone. Because AtAER does not react with non-RCS carbonyls [87], the suppression of them in the AER-OE plants must have been an indirect effect of the transgene. A possible explanation of this effect is that there was a long chain RCS(s) that decomposed to generate various short carbonyls and AER scavenged this precursor RCS. Such a long chain RCS could have escaped from our carbonyl analyses because we have optimized the extraction method for hydrophilic RCS such as acrolein and HNE [85,86]. The second intriguing fact was that the basal levels of RCS and non-RCS carbonyls in cells were not affected by the overexpression of AER in the cytosol. This indicates that the constitutive RCS and non-RCS are generated in compartments different from the cytosol, and cells tolerate such levels of carbonyls. Also, it is suggested that the suppression of increases in the cytosolic carbonyl diminished RCS damage and RCS signaling.

AOR from *A. thaliana* (AtAOR, encoded by the gene At1g23740) recognizes RCS such as 3-buten-2-one, 4-hexen-3-one, 1-penten-3-one, crotonaldehyde and acrolein as substrates [106]. The *AOR* knockout mutant of *A. thaliana*, when illuminated in the presence of methylviologen, accumulated higher level of acrolein and suffered severer damage in photosystem I than did wild type [111]. This can be interpreted as the enhancement of a toxic effect of acrolein. Another phenotype of the knockout mutant was the suppression of carbon catabolism during night [112]. The mutant showed a significantly lower level of phosphoenolpyruvate carboxylase activity and incomplete degradation of starch in chloroplasts during night. The results suggest a critical importance of certain carbonyls in the regulation of carbonyls, and further investigation to identify the involved carbonyl species is expected. 

#### 5.2.2. Glutathione Transferase (GST): Conjugation of RCS with GSH

GST catalyzes the binding of GSH with an electrophilic compound to form a conjugate (Figure 4). This is the first step of detoxification. The GSH-electrophile conjugate is subsequently degraded via several enzyme reactions [96,97]. GST isozymes in plants are classified into at least 13 classes, of which Tau (U) class is the largest member [113]. Each isozyme has a different substrate (i.e., electrophile) specificity [114]. Also, most of the GST isozymes have a GSH-dependent peroxidase activity [114], specifically, the reduction of LOOH to a corresponding alcohol using GSH as the electron donor. 

GST has been known as a general detoxification enzyme. A number of reports are available that demonstrate that overexpression of certain GST genes improved stress tolerance of the plants [115,116,117], and references therein], but it remains unclear which toxic compounds were scavenged in such transgenic plants. Only several GST isozymes had been known to have the activity to detoxify RCS, such as those in sorghum [118], grapevine [119], spinach and *A. thaliana* [61], specifically, they catalyze the conjugation of RCS with GSH. A recent comprehensive assay of 21 GSTU isozymes of *A. thaliana* revealed that at least 11 of them recognize RCS as a substrate, suggesting that RCS are major physiological substrates for GST isozymes [60].

One of the insights obtained from the comprehensive analysis of substrate specificity in GSTU isozymes was that similarity of substrate recognition pattern is not necessarily correlated with the sequence-based phylogeny of isozymes, as follows. Ten AtGSTU isozymes found to react with acrolein were distributed in three GSTU subclasss of the four, the classification based on the amino acid sequence homology, and each subclass in the above three also contains isozymes that are imcompatible with acrolein. AtGSTU20, the closest to AtGSTU19 (88.48% amino acid identity) in the phylogenetic tree, does not react with acrolein at all [60]. Six isozymes able to detoxify HNE were found in two subclasses, each of which contain HNE incompatible isozymes, too. The GSTU25 isozyme was specific to (*E*)-2-hexenal and crotonaldehyde, but it did not react with either acrolein or HNE. For HNE, GSTU17 and GSTU18 were the most efficient scavengers. It is also noticable that distinct GST isozyme has relatively narrow specificity for RCS. This gives contrast to rather broad substrate specificities observed for reductases such as AtAER [76,87] and AKR [120].

Interestingly, the gene of the isozyme AtGSTU19, an apparently important isozyme showing the highest activity for acrolein with a *K*_m_ value as low as 30 μM [60], is constitutively expressed at relatively high level in almost all tissues and it responded to induced by only limited kinds of stress stimuli [121]. This implies the necessity of the acrolein scavenging reaction(s) in the whole body of the plant. On the other hand, knocking out of the AtGSTU17 gene resulted in a higher GSH and ABA levels and tolerance against drought and salt stress [115]. Based on a high activity of AtGSTU17 for HNE and acrolein [60], it is likely that these RCS are involved in the regulation of the biosynthesis of GSH and ABA. Analysis of carbonyl content changes made by the overexpression/knocking out of these GSTU isozymes will give us deep insights into the roles of RCS under non-stressed and stressed conditions.

## 6. Perspectives

As reviewed above, the physiological importance of RCS as mediators of ROS signals is now established for PCD, senescence of silique, heat shock-induced gene regulation, ABA-induced stomata closure and auxin-induced LR formation (Figure 1). Considering the critical role of ROS in various physiological situations, we can expect that the action of RCS to determine cell fate is not limited to the phenomena above. For judging the involvement of RCS in an event, the following criteria are useful; (i) upstream stimulus causes the increase in RCS levels prior to the final outcome phenomenon, (ii) suppression of RCS by scavengers diminishes the ROS-induced response, and (iii) RCS evoke a similar response to that induced by ROS. 

Thus, we are now at the entrance of an unexplored research field of ‘RCS physiology in plants’. The exploration will bring us rich knowledge of the dynamic action of the reactive species in determining cell’s life and death. Such fundamental knowledge can be applied to the development of advanced strategies for improving crop yields. At the end of this review, we present several challenges we face now. Although there are technical difficulties in solving the problems, we will see significant progress in coming years. The more researchers join, the farther and broader will we able to explore.

### 6.1. Contribution of Distinct Carbonyl Species

The carbonyl analysis enabled us to prove the increases of RCS in early stages of plant’s response to ROS stimuli. The RCS commonly observed to increase early were acrolein and HNE. Other RCS, e.g., crotonaldehyde, (*E*)-2-pentenal and (*E*)-2-hexenal, have been also increased by ROS stimuli, and furthermore, non-RCS carbonyls such as formaldehyde, acetaldehyde, butyraldehyde and *n*-hexanal were found to increase. Among these carbonyl species, acrolein and HNE are probably the most critical species because both of them evoke physiological responses at low concentrations [88,93]. Other RCS are apparently less active to induce cellular responses and therefore physiological effects of the chemical group designated as RCS or RES-oxylipins are not uniform. In addition, several non-RCS carbonyls also exerted similar effects that were evoked by RCS, at a concentration same to that of crotonaldehyde, a relatively mild RCS. As for initiating PCD, it has been found that these less reactive carbonyls also contribute to the result to a 1/3-1/10 extent of acrolein or HNE, suggesting that not only the *α*,*β*-unsaturated carbonyls but also several saturated carbonyls do participate in the ROS signaling [88]. To make things more complicated, the effect to enhance the auxin signaling [93] was observed RCS and only several species of non-RCS carbonyls, indicating that non-RCS carbonyls also are not uniform in their functions. As our knowledge comes to this stage, there may be a question about the validity of the grouping of RCS or RES-oxylipins. For the moment, however, there is not an appropriate term that inclusively express these physiologically relevant carbonyl compounds. The term RCS is therefore just expedient to express a group of highly reactive and biologically active carbonyls represented by acrolein and HNE, until more detailed study of the roles of distinct carbonyls will have been developed. 

### 6.2. Regulation of Distinct Carbonyl Species

Some RCS scavenging enzymes, e.g., GSTU, show narrow specificities to carbonyls and others, e.g., AKR and ALDH, broad. As seen in GSTU isozymes, proteins have such potential to strictly distinguish small molecules even though they are “reactive”. Therefore, both the narrowness and the broadness of substrate specificity must be evolutional consequences of physiological requirements. When comprehensive substrate specificity analysis of every isozyme in each enzyme class is combined with the data of expression pattern (tissue, subcellular localization and induction), it will be an important piece of the whole picture of RCS physiology in plants.

### 6.3. Action Mechanisms of RCS Signal

As discussed above, even among “reactive” carbonyl species, the biological effects are strikingly different. Such functional difference may be most probably the result of the specificity of the receptor/sensor proteins, though it can be partially ascribed to the differences in the chemical properties among distinct carbonyl species [122]. Our knowledge about RCS receptors/sensors is still only limited, but these putative receptor/sensor proteins appear to have more strict specificities than expected from the collective term RCS, which could imply non-specific reactions.

In plants, the only identified RCS receptor is cyclophilin 20–2, which is regulated by OPDA [66], but the reactivity of this protein with various RCS has not been examined yet. For PCD, it was found that C3LP is activated directly by acrolein and HNE [65]. To investigate the activation mechanism further, the protein responsible for this activity should be identified first; there are two candidate proteins, i.e., cathepsin B [123] and the subunit PBA1 of 26S proteasome [124]. For the auxin signaling for LR formation, one of the most likely candidates of RCS sensor is TIR1 [93]. When this is verified, then its responses to non-RCS carbonyls should be analyzed to answer the question. There has been a critical question about the mechanism how ROS, broadly reacting species, transmit distinct physiological signals [125,126]. One of the keys to this is the specificity of the signal receptors. Investigation of RCS receptor specificity may provide a great help to solve this problem.

## Figures and Tables

**Table 1:** List of plant proteins that are modified with RCS or inactivated by RCS. From the proteomic analyses [52,53], representative proteins are extracted. SU, subunit.

Compartment	Protein	RCS	Effect	Ref
**Mitochondrion**	GDC H-SU and other lipoate enzymes	HNE	Modified, Inactivated	[63]
	Alternative oxidase	HNE	Inactivated	[64]
	Succinate dehydrogenase *α-*SU ATP synthase *β*-SU Pyruvate dehydrogenase E1 *β*-SU Elongation factor Tu Voltage-dependent anion channel Adenine nucleotide translocator	HNE	Modified	[52]
**Chloroplast**	OEC33 LHCII	MDA	Modified	[51]
	Phosphoribulokinase Glyceraldehyde-3-phosphate dehydrogenase Fructose-1,6-bisphosphatase	acrolein	Inactivated	[13]
	Cyclophilin 20-3	HNE	Modified	[53]
		OPDA	Modified, Activated	[66]
**Cytosol**	Triosephosphate isomerase Cysteine synthase Ascorbate peroxidase Heat shock cognate 70 kDa protein 3	HNE	Modified	[53]
	C1LP, C3LP	acrolein, HNE	Activated	[65]
**Peroxisome**	Nitrile-specifier protein 5 Gly-rich RNA binding protein 7 Nucleotide diphosphate kinase	HNE	Modified	[53]
**Apoplast**	Germin-like protein subfamily 3 number 1 Peroxidase 34	HNE	Modified	[53]

## References

[1] Foyer C.H., Noctor G. (2003). Redox sensing and signalling associated with reactive oxygen in chloroplasts, peroxisomes and mitochondria. Physiol. Plant..

[2] Karpinski S., Reynolds H., Karpinska B., Wingsle G., Creissen G., Mullineaux P. (1999). Systemic signaling and acclimation in response to excess excitation energy in *Arabidopsis*. Science.

[3] Mittler R. (2017). ROS are good. Trends Plant Sci..

[4] Pei Z.M., Murata Y., Benning G., Thomine S., Klüsener B., Allen G.J., Grill E., Schroeder J.I. (2000). Calcium channels activated by hydrogen peroxide mediate abscisic acid signalling in guard cells. Nature.

[5] Orman-Ligeza B., Parizot B., de Rycke R., Fernandez A., Himschoot E., Breusegem F.V., Bennett M.J., Périlleux C., Beeckman T., Draye X. (2016). RBOH-mediated ROS production facilitates lateral root emergence in Arabidopsis. Development.

[6] Li S., Xue L., Xu S., Feng H., An L. (2007). Hydrogen peroxide involvement in formation and development of adventitious roots in cucumber. Plant Growth Regul..

[7] Bethke P.C., Jones R.L. (2001). Cell death of barley aleurone protoplasts is mediated by reactive oxygen species. Plant J..

[8] Torres M.A., Jones J.D.G., Dangl J.L. (2005). Pathogen-induced, NADPH oxidase–derived reactive oxygen intermediates suppress spread of cell death in *Arabidopsis thaliana*. Nat. Genet..

[9] Mano J. (2012). Reactive carbonyl species: Their production from lipid peroxides, action in environmental stress, and the detoxification mechanism. Plant Physiol. Biochem..

[10] Asada K. (2006). Production and scavenging of reactive oxygen species in chloroplasts and their functions. Plant Physiol..

[11] Mène-Saffrané L., Davoine C., Stolz S., Majcherczyk P., Farmer E.E. (2007). Genetic removal of tri-unsaturated fatty acids suppresses developmental and molecular phenotypes of an *Arabidopsis* tocopherol-deficient mutant. J. Biol. Chem..

[12] Schauenstein E., Esterbauer H., Zollner H. (1977). Aldehyde in Biological Systems: Their Natural Occurrence and Biological Activities.

[13] Mano J., Miyatake F., Hiraoka E., Tamoi M. (2009). Evaluation of the toxicity of stress-related aldehydes to photosynthesis in chloroplasts. Planta.

[14] Yin L., Mano J., Wang S., Tsuji W., Tanaka K. (2010). The involvement of lipid peroxide-derived aldehydes in aluminum toxicity of tobacco roots. Plant Physiol..

[15] Mano J., Tokushige K., Mizoguchi K., Fujii H., Khorobrykh S. (2010). Accumulation of lipid peroxide-derived, toxic *α*,*β*,-unsaturated aldehyde (*E*)-2-pentenal, acrolein and (*E*)-2-hexenal in leaves under photoinhibitory illumination. Plant Biotechnol..

[16] Farmer E.E., Mueller M.J. (2013). ROS-mediated lipid peroxidation and RES-activated signaling. Annu. Rev. Plant Biol..

[17] Mueller M.J., Berger S. (2009). Reactive electrophile oxylipins: Pattern recognition and signalling. Phytochemistry.

[18] Liebler D.C. (2008). Protein damage by reactive electrophiles: Targets and consequences. Chem. Res. Toxicol..

[19] Hoque T.S., Hossain M.A., Mostofa M.G., Burritt D.J., Fujita M., Tran L.-S.P. (2016). Methylglyoxal: An emerging signaling molecule in plant abiotic stress responses and tolerance. Front. Plant Sci..

[20] Esterbauer H., Schauer R., Zollner J.H. (1991). Chemistry and biochemistry of 4-hydroxynonenal, malondialdehyde and related aldehydes. Free Rad. Biol. Med..

[21] West J.D., Marnett L.J. (2006). Endogenous reactive intermediates as modulators of cell signaling and cell death. Chem. Res. Toxicol..

[22] Wible R.S., Sutter T.R. (2017). Soft cysteine signaling network: The functional significance of cysteine in protein function and the soft acids/bases thiol chemistry that facilitates cysteine modification. Chem. Res. Toxicol..

[23] Schopfer F.J., Cipollina C., Freeman B.A. (2011). Formation and signaling actions of electrophilic lipids. Chem. Rev..

[24] Yalcinkaya T., Uzilday B., Ozgur R., Turkan I., Mano J. (2019). Lipid peroxidation-derived reactive carbonyl species (RCS): Their interaction with ROS and cellular redox during environmental stresses. Environ. Exp. Bot..

[25] D’Alessandro S., Havaux M. (2019). Sensing β-carotene oxidation in photosystem II to master plant stress tolerance. N. Phytol..

[26] Grosch W., Chan H.W.-S. (1987). Reactions of hydroperoxides—Products of low molecular weight. Autoxidation of Unsaturated Lipids.

[27] Halliwell B., Gutteridge J.M.C. (2015). Free Radicals in Biology and Medicine.

[28] Blée E. (1998). Phytooxylipins and plant defense reactions. Prog. Lipid Res..

[29] Schaller F. (2001). Enzymes of the biosynthesis of octadecanoid-derived signalling molecules. J. Exp. Bot..

[30] Matsui K. (2006). Green leaf volatiles: Hydroperoxide lyase pathway of oxylipin metabolism. Curr. Opin. Plant Biol..

[31] Triantaphylidès C., Krischke M., Hoeberichts F.A., Ksas B., Gresser G., Havaux M., Van Breusegem F., Mueller M.J. (2008). Singlet oxygen is the major reactive oxygen species involved in photooxidative damage to plants. Plant Physiol..

[32] Boeglin W.E., Itoh A., Zheng Y., Coffa G., Howe G.A., Brash A.R. (2008). Investigation of substrate binding and product stereochemistry issues in two linoleate 9-lipoxygenases. Lipids.

[33] Bannenberg G., Martínez M., Hamberg M., Castresana C. (2009). Diversity of the Enzymatic activity in the lipoxygenase gene family of *Arabidopsis thaliana*. Lipids.

[34] Kishimoto K., Matsui K., Ozawa R., Takabayashi J. (2005). Volatile C6-aldehydes and allo-ocimene activate defense genes and induce resistance against *Botrytis cinerea* in *Arabidopsis thaliana*. Plant Cell Physiol..

[35] Shiojiri K., Kishimoto K., Ozawa R., Kugimiya S., Urashimo S., Arimura G., Horiuchi J., Nishioka T., Matsui K., Takabayashi J. (2006). Changing green leaf volatile biosynthesis in plants: An approach for improving plant resistance against both herbivores and pathogens. Proc. Natl. Acad. Sci. USA.

[36] Sugimoto K., Matsui K., Iijima K., Akakabe Y., Muramoto S., Ozawa R., Uefune M., Sasaki R., Alamgir K.Md., Akitake S. (2014). Intake and transformation to a glycoside of (*Z*)-3-hexenol from infested neighbors reveals a mode of plant odor reception and defense. Proc. Natl. Acad. Sci. USA.

[37] Hammarberg T., Provost P., Persson B., Rådmark O. (2000). The N-terminal domain of 5-lipoxygenase binds calcium and mediates calcium stimulation of enzyme activity. J. Biol. Chem..

[38] Kulkarni S., Das S., Funk C.D., Murray D., Cho W. (2002). Molecular basis of the specific subcellular localization of the C2-like domain of 5-lipoxygenase. J. Biol. Chem..

[39] Mochizuki S., Matsui K. (2018). Green leaf volatile-burst in Arabidopsis is governed by galactolipid oxygenation by a lipoxygenase that is under control of calcium ion. Biochem. Biophys. Res. Commun..

[40] Montillet J.-L., Chamnongpol S., Rustérucci C., Dat J., van de Cotte J., Agnel J.-P., Battesti C., Inzé D., Van Breusegem F., Triantaphylidès C. (2005). Fatty acid hydroperoxides and H_2_O_2_ in the execution of hypersensitive cell death in tobacco leaves. Plant Physiol..

[41] Montillet J.-L., Cacas J.-L., Lionel Garnier L., Montané M.H., Douki T., Bessoule J.-J., Polkowska-Kowalczyk L., Maciejewska U., Agnel J.-P., Vial A. (2004). The upstream oxylipin profile of *Arabidopsis thaliana*: A toolto scan for oxidative stresses. Plant J..

[42] Schneider C., Tallman K.A., Porter N.A., Brash A.R. (2001). Two distinct pathways of formation of 4-hydroxynonenal. J. Biol. Chem..

[43] Lee S.H., Oe T., Blair I.A. (2001). Vitamin C-induced decomposition of lipid hydroperoxides to endogenous genotoxins. Science.

[44] Mochizuki S., Sugimoto K., Koeduka T., Matsui K. (2016). Arabidopsis lipoxygenase 2 is essential for formation of green leaf volatiles and five-carbon volatiles. FEBS Lett..

[45] Kunishima M., Yamauchi Y., Mizutani M., Kuse M., Takikawa H., Sugimoto Y. (2016). Identification of (*Z*)-3:(*E*)-2-hexenal isomerases essential to the production of the leaf aldehyde in plants. J. Biol. Chem..

[46] Spyropoulou E.A., Dekker H.L., Steemers L., van Maarseveen J.H., de Koster C.G., Haring M.A., Schuurink R.C., Allmann S. (2017). Identification and Characterization of (3*Z*):(2*E*)-Hexenal Isomerases from Cucumber. Front. Plant Sci..

[47] Gardner H.W. (2016). Formation of (2*E*)-4-hydroxyl-2-nonenal and (2*E*)-4-hydroxy-2-hexenal by plant enzymes: A review suggests a role in the physiology in plants. Adv. Enzyme Res..

[48] Mano J., Khorobrykh S., Matsui K., Iijima Y., Sakurai N., Suzuki H., Shibata D. (2014). Acrolein is formed from trienoic fatty acids in chloroplasts: A targeted metabolomics approach. Plant Biotechnol..

[49] Roach T., Baur T., Stöggl W., Krieger-Litszkay A. (2017). *Chlamydomonas reinhardtii* responding to high light: A role for 2-propenal (acrolein). Physiol. Plant..

[50] Roach T., Stöggl W., Baur T., Kranner I. (2018). Distress and eustress of reactive electrophiles and relevance to light stress acclimation via stimulation of thiol/disulfide-based defences. Free Rad. Biol. Med..

[51] Yamauchi Y., Furutera A., Seki K.Y., Toyoda Y., Tanaka K., Sugimoto Y. (2008). Malondialdehyde generated from peroxidized linoleic acid causes protein modification in heat-stressed plants. Plant Physiol. Biochem..

[52] Winger A.M., Taylor N.L., Heazlewood J.L., Day D.A., Miller A.H. (2007). The cytotoxic lipid peroxidation product 4-hydroxy-2-nonenal covalently modifies a selective range of proteins linked to respiratory function in plant mitochondria. J. Biol. Chem..

[53] Mano J., Nagata M., Okamura S., Shiraya T., Mitsui T. (2014). Identification of oxidative-modified proteins in salt-stressed Arabidopsis: A carbonyl-targeted proteomics approach. Plant Cell Physiol..

[54] Esterbauer H., Zollner H., Scholtz N. (1975). Reaction of glutathione with conjugated carbonyls. Zeitschrift für Naturforschung C.

[55] Møller I.M., Jensen P.E., Hansson A. (2007). Oxidative modifications to cellular components in plants. Annu. Rev. Plant Biol..

[56] Bachi A., Dalle-Donne I., Scaloni A. (2013). Redox proteomics: Chemical principles, methodological approaches and biological/biomedical promises. Chem. Rev..

[57] LoPachin R.M., Gavin T., Peterson D.R., Barber D.S. (2009). Molecular mechanisms of 4-hydroxyn-2-nonenal and acrolein toxidity: Nucleophilic targets and adduct formation. Chem. Research Toxicol..

[58] Rudolph T.K., Freeman B.A. (2009). Transduction of redox signaling by electrophile-protein reactions. Sci. Signal..

[59] Arnette D., Quillin A., Gledenhuys W.J., Menze M.A., Konkle M. (2019). 4-Hydroxynonenal and 4-ocononenal differentially bind to the redox sensor mitoNEET. Chem. Res. Toxicol..

[60] Mano J., Kanameda S., Kuramitsu R., Matsuura N., Yamauchi Y. (2019). Detoxification of reactive carbonyl species by glutathione transferase Tau isozymes. Front. Plant Sci..

[61] Mano J., Ishibashi A., Muneuchi H., Morita C., Sakai H., Biswas S., Kitajima S. (2017). Acrolein-detoxifying isozymes of glutathione transferase in plants. Planta.

[62] Pampola R. (2011). Advanced lipoxidation end-products. Chem. Biol. Interact..

[63] Taylor N.L., Day D.A., Millar A.H. (2002). Environmental stress causes oidative damage to plant mitochondria leading to inhibition of glycine decarboxylase. J. Biol. Chem..

[64] Winger A.M., Millar A.H., Day D.A. (2005). Sensitivity of plant mitochondrial terminal oxidases to the lipid peroxidation product 4-hydroxy-2-nonenal (HNE). Biochem. J..

[65] Biswas M.S., Mano J. (2016). Reactive carbonyl species activate caspase-3-like protease to initiate programmed cell death in plants. Plant Cell Physiol..

[66] Park S.-W., Li W., Viehhauser A., He B., Kim S., Nilsson A.K., Andersson M.X., Kittle J.D., Ambavaram M.M.R., Luan S. (2013). Cyclophilin 20-3 relays a 12-oxo-phytodienoic acid signal during stress responsive regulation of cellular redox homeostasis. Proc. Natl. Acad. Sci. USA.

[67] Viestra R.D. (2003). The ubiquitin/26S proeasome pathway, the complex last chapter in the life of many plant proteins. Trends Plant Sci..

[68] Zheng J., Bizzozero O. (2010). Reduced proteasomal activity contributes to the accumulation of carbonylated proteins in chromic experimental autoimmune encephalomyelitis. J. Neurochem..

[69] Kurepa J., Toh-e A., Smalle J.A. (2008). 26S proteasome regulatory particle mutants have increased oxidative stress tolerance. Plant J..

[70] Smakowska E., Czarna M., Janska H. (2014). Mitochondrial ATP-dependent proteases in protection against accumulation of carbonylated proteins. Mitochondrion.

[71] Tsuchiya Y., Okada G., Kobayashi S., Chikuma T., Hojo H. (2011). 4-Hydroxy-2-nonenal-modified glyceraldehyde-3-phosphate dehydrogenase is degraded by cathepsin G in rat neutrophils. Oxid. Med. Cell. Longev..

[72] Weber H., Chételat A., Reymond P., Farmer E.E. (2004). Selective and powerful stress gene expression in *Arabidopsis* in response to malondialdehyde. Plant J..

[73] Reynolds T. (1977). Comparative effects of aliphatic compounds on inhibition of lettuce fruit germination. Ann. Bot..

[74] Alméras E., Stolz S., Vollenweider S., Reymond P., Mène-Saffrané L., Farmer E.E. (2003). Reactive electrophile species activate defense gene expression in *Arabidopsis*. Plant J..

[75] Matsui K., Sugimoto K., Mano J., Ozawa R., Takabayashi J. (2012). Differential metabolism of green leaf volatiles in injured and intact parts of a wounded leaf meet distinct ecophysiological requirements. PLoS ONE.

[76] Mano J., Belles-Boix E., Babiychuk E., Inzé D., Torii Y., Hiraoka E., Takimoto K., Slooten L., Asada K., Kushnir S. (2005). Protection against photooxidative injury of tobacco leaves by 2-alkenal reductase. Detoxication of lipid peroxide-derived reactive carbonyls. Plant Physiol..

[77] Millar A.H., Leaver C.L. (2000). The cytotoxic lipid peroxidation product, 4-hydroxy-2-nonenal, specifically inhibits decarboxylating dehydrogenases in the matrix of plant mitochondria. FEBS Lett..

[78] Shimakawa G., Iwamoto T., Mabuchi T., Saito R., Yamamoto H., Amako K., Sugimoto T., Makino A., Miyake C. (2013). Acrolein, an *α*,*β*-unsaturated carbonyl, inhibits both growth and PSII activity in the cyanobacterium *Synechocystis* sp. PCC 6803. Biosci. Biotechnol. Biochem..

[79] Bate N.J., Rothstein S.J. (1998). C6-volatiles derived from the lipoxygenase pathway induce a subset of defense-related genes. Plant J..

[80] Yamauchi Y., Kunishima M., Mizutani M., Sugimoto Y. (2015). Reactive short-chain leaf volatiles act as powerful inducers of abiotic stress-related gene expression. Sci. Rep..

[81] Yalcinkaya T., Uzilday B., Ozgur R., Turkan I. (2019). The roles of reactive carbonyl species in induction of antioxidant defence and ROS signalling in extreme halophytic model *Eutrema parvulum* and glycophytic model *Arabidopsis thaliana*. Exp. Environ. Bot..

[82] Taki N., Sasaki-Sekimoto Y., Obayashi T., Kikuta A., Kobayashi K., Ainai T., Yagi K., Sakurai N., Suzuki H., Masuda T. (2005). 12-Oxo-phytodienoic acid triggers expression of a distinct set of genes and plays a role in wound-induced gene expression in arabidopsis. Plant Physiol..

[83] Eckardt N.A. (2008). Oxylipin signaling in plant stress responses. Plant Cell.

[84] Stotz H.U., Mueller S., Zoeller M., Mueller M.J., Berger S. (2013). TGA transcription factors and jasmonate-independent COI1 signalling regulate specific plant responses to reactive oxylipins. J. Exp. Bot..

[85] Matsui K., Sugimoto K., Kakumyan P., Khorobrykh S.A., Mano J., Armstrong D. (2009). Volatile oxylipins formed under stress in plants. Lipidomics, Methods in Molecular Biology.

[86] Mano J., Biswas M.S., De Gara L., Locato V. (2018). Analysis of reactive carbonyl species generated under oxidative stress. Plant Programmed Cell Death: Methods and Protocols.

[87] Mano J., Torii Y., Hayashi S., Takimoto K., Matsui K., Nakamura K., Inzé D., Babyichuk E., Kushnir S., Asada K. (2002). The NADPH:quinone oxidoreductase P1-ζ-cryatallin in Arabidopsis catalyzes the *α*,*β*-hydrogenation of 2-alkenals: Detoxication of the lipid peroxide-derived reactive aldehydes. Plant Cell Physiol..

[88] Biswas M.S., Mano J. (2015). Lipid peroxide-derived short-chain carbonyls mediate hydrogen peroxide-induced and salt-induced programmed cell death in plants. Plant Physiol..

[89] Kai H., Hirashima K., Matsuda O., Ikegami H., Winkelmann T., Nakahara T., Iba K. (2012). Thermotolerant cyclamen with reduced acrolein and methyl vinyl ketone. J. Expt. Bot..

[90] Srivastava S., Brychkova C., Yarmolinsky D., Soltabayeva A., Samani T., Sagi M. (2017). Aldehyde oxidase 4 plays a critical role in delaying silique senescence by catalyzing aldehyde detoxification. Plant Physiol..

[91] Islam M.M., Ye W., Matsushima D., Munemasa S., Okuma E., Nakamura Y., Biswas M.S., Mano J., Murata Y. (2016). Reactive carbonyl species mediate abscisic acid signaling in guard cells. Plant Cell Physiol..

[92] Islam M.M., Ye W., Matsushima D., Rhaman M.S., Munemasa S., Okuma E., Nakamura Y., Biswas M.S., Mano J., Murata Y. (2019). Reactive carbonyl species function as signal mediators downstream of H_2_O_2_ production and regulate [Ca^2+^]_cyt_ elevation in ABA signal pathway in Arabidopsis guard cells. Plant Cell Physiol..

[93] Biswas M.S., Fukaki H., Mori I.C., Nakahara K., Mano J. (2019). Reactive oxygen species and reactive carbonyl species constitute a feed-forward loop in the auxin signaling for lateral root formation. Plant J..

[94] Ramel F., Birtic S., Ginies C., Soubigou-Taconnat L., Triantaphylidès C., Havaux M. (2012). Carotenoid oxidation products are stress signals that mediate gene responses to singlet oxygen in plants. Proc. Natl. Acad. Sci. USA.

[95] Yin L., Mano J., Tanaka K., Wang S., Zhang M., Deng X., Zhang S. (2017). High level of reduced glutathione contributes to detoxification of lipid peroxide-derived reactive carbonyl species in transgenic Arabidopsis overexpressing glutathione reductase under aluminum stress. Physiol. Plant..

[96] Ohkama-Ohtsu N., Zhao P., Xiang C., Oliver D.J. (2007). Glutathione conjugates in the vacuole are degraded by γ-glutamyl transpeptidase GGT3 in Arabidopsis. Plant J..

[97] Grzam A., Martin M.N., Hell R., Meyer A.J. (2007). γ-Glutamyl transpeptidase GGT4 initiates vacuolar degradation of glutathione *S*-conjugates in *Arabidopsis*. FEBS Lett..

[98] Aldini G., Carini M., Beretta C., Bradamante S., Facino R.M. (2002). Carnosine is a quencher of 4-hydroxy-nonenal: Through what mechanism of reaction?. Biochem. Biophys. Res. Commun..

[99] Carini M., Aldini G., Beretta G., Arlandini E., Facino R.M. (2003). Acrolein-sequestering ability of endogenous dipeptides: Characterization of carnosine and homocarnosine/acrolein adducts by electrospray ionization tandem mass spectrometry. J. Mass Spectrom..

[100] Zhu Q., Zheng Z.-P., Cheng K.-W., Wu J.-J., Zhang S., Tang Y.S., Sze K.-H., Chen J., Chen F., Wang M. (2009). Natural polyphenols as direct trapping agents of lipid peroxidation-derived acrolein and 4-hydroxy-trans-2-nonenal. Chem. Res. Toxicol..

[101] Colzani M., Regazzoni L., Criscuolo A., Baron G., Carini M., Vistoli G., Lee Y.-M., Han S.-I., Aldini G., Yeum K.-J. (2018). Isotopic labelling for the characterisation of HNE-sequestering agents in plant-based extracts and its application for the identification of anthocyanidins in black rice with giant embryo. Free Rad. Res..

[102] Sengupta D., Naik D., Reddy A.R. (2015). Plant aldo-keto reductases (AKRs) as multi-tasking soldiers involved in diverse plant metabolic processes and stress defense: A structure-function update. J. Plant Physiol..

[103] Kirch H.-H., Bartels D., Wei Y., Schnable P.S., Wood A.J. (2004). The ALDH gene superfamily of Arabidopsis. Trends Plant Sci..

[104] Brocker C., Vasiliou M., Carpenter S., Carpenter C., Zhang Y., Wang X., Kotchoni S.O., Wood A.W., Kirch H.-H., Kopecˇny´ D. (2012). Aldehyde dehydrogenase (ALDH) superfamily in plants: Gene nomenclature and comparative genomics. Planta.

[105] Stiti N., Missihoun T.D., Kotchoni S.O., Kirch H.-H., Bartels D. (2011). Aldehyde dehydrogenases in Arabidopsis thaliana: Biochemical requirements, metabolic pathways, and functional analysis. Front. Plant Sci..

[106] Yamauchi Y., Hasegawa A., Taninaka A., Mizutani M., Sugimoto Y. (2011). NADPH-dependent reductases involved in the detoxification of reactive carbonyls in plants. J. Biol. Chem..

[107] Nordling E., Jörnvall H., Persson B. (2002). Medium-chain dehydrogenases/reductases (MDR) Family characterizations including genome comparisons and active site modelling. FEBS J..

[108] Youn B., Kim S.-J., Moinuddin S.G.A., Lee C., Bedgar D.L., Harper A.R., Davin L.B., Lewis N.G., Kang C.H. (2006). Mechanistic and structural studies of apoform, binary, and ternary complexes of the arabidopsis alkenal double bond reductase At5g16970. J. Biol. Chem..

[109] Koeduka T., Watanabe B., Suzuki S., Hiratake J., Mano J., Yazaki K. (2011). Characterization of raspberry ketone/zongerone synthase, catalyzing the alpha,beta-hydrogenation of phenylbutanones in raspberry fruits. Biochem. Biophys. Res. Commun..

[110] Papdi C., Ábrahám E., Joseph M.P., Popescu C., Koncz C., Szabados L. (2008). Functional identification of Arabidopsis stress regulatory genes using the controlled cDNA overexpression system. Plant Physiol..

[111] Yamauchi Y., Hasegawa A., Mizutani M., Sugimoto Y. (2012). Chloroplastic NADPH-dependent alkenal/one oxidoreductase contributes to the detoxification of reactive carbonyls produced under oxidative stress. FEBS Lett..

[112] Takagi D., Ifuku K., Ikeda K., Inoue K.I., Park P., Tamoi M., Inoue H., Sakamoto K., Saito R., Miyake C. (2016). Suppression of chloroplastic alkenal/one oxidoreductase represses the carbon catabolic pathway in Arabidopsis leaves during night. Plant Physiol..

[113] Monticolo F., Colantuono C., Chusano M.L. (2017). Shaping the evolutionary tree of green plants: Evidence from the GST family. Sci. Rep..

[114] Dixon D.P., Hawkins T., Hussey P.J., Edwards R. (2009). Enzyme activities an subcellular localization of members of the Arabidopsis glutathione transferase superfamily. J. Exp. Bot..

[115] Chen J.H., Jiang H.-W., Hsieh E.-J., Chen H.-Y., Chien C.-T., Hsieh H.-L., Lin T.-P. (2012). Drought and salt stress tolerance of an Arabidopsis glutathione *S*-transferase U17 knockout mutant are attributed to the combined effect of glutathione and abscisic acid. Plant Physiol..

[116] Xu J., Tian Y.-S., Xing X.-J., Peng R.-H., Zhu B., Gao J.-J., Yao Q.-H. (2016). Over-expression of *AtGSTU19* provides tolerance to salt, drought and methyl viologen stresses in *Arabidopsis*. Physiol. Plant..

[117] Sharma R., Sahoo A., Devendran R., Jain M. (2014). Over-expression of a rice Tau class glutathione-S-transferase gene improves tolerance to salinity and oxidative stresses in Arabidopsis. PLoS ONE.

[118] Gronwald J.W., Plaisance K.L. (1998). Isolation and characterization of glutathione *S*-transferase isozymes from sorghum. Plant Physiol..

[119] Kobayashi H., Takase H., Suzuki Y., Tanzawa F., Tanaka R., Fujita K., Kohno M., Mochizuki M., Suzuki S., Konno T. (2011). Environment stress enhances biosynthesis of flavor precursors, *S*-3-(hexan-1-ol)-glutathione and *S*-3-(hexan-1-ol) L-cysteine, in grapevine through glutathione *S*-transferase activation. J. Exp. Bot..

[120] Simpson P.J., Tantitadapitak C., Reed A.M., Mather O.C., Bunce C.M., White S.A., Ride J.P. (2009). Characterization of two novel aldo-keto reductases from *Arabidopsis*: Expression patterns, broad substrate specificity, and an open active-site structure suggest a role in toxicant metabolism following stress. J. Mol. Biol..

[121] Kilian J., Whitehead D., Horak J., Wanke D., Weinl S., Batistic O., D’Angelo C., Bornberg-Bauer E., Kudla J., Harter K. (2007). The AtGenExpress global stress expression data set: Protocols, evaluation and model data analysis of UV-B light, drought and cold stress responses. Plant J..

[122] LoPachin R.M., Gavin T., DeCaprio A., Barber D.S. (2012). Application of the hard and soft, acids and bases (HSAB) theory to toxicant-target interactions. Chem. Res. Toxicol..

[123] Ge Y., Cai Y.-M., Bonneau L., Rotari V., Danon A., MacKenzie E.A., McLellan H., Mach L., Gallois P. (2016). Inhibition of cathepsin B by caspase-3 inhibitors blocks programmed cell death in Arabidopsis. Cell Death Differ..

[124] Hatsugai N., Iwasaki S., Tamura K., Kondo M., Fuji K., Ogasawara K., Nishimura M., Hara-Nishimura I. (2009). A novel membrane fusion-mediated plant immunity against bacterial pathogens. Genes Dev..

[125] Møller I.M., Sweetlove L.J. (2010). ROS signaling—Specificity is required. Trends Plant Sci..

[126] Higdon A., Diers A.R., Oh J.Y., Lander A., Darley-Usmar M. (2012). Cell signalling by reactive lipid species: A new concepts and molecular mechanisms. Biochem. J..

